# Monocular Vision Loss: A Rare Cause

**DOI:** 10.5811/cpcem.2019.6.43224

**Published:** 2019-03-27

**Authors:** David Lane, Kaila Pomeranz, Shannon Findlay, Daniel Miller

**Affiliations:** University of Iowa Hospitals and Clinics, Department of Emergency Medicine, Iowa City, Iowa

## Abstract

A 62-year-old woman with a history of metastatic breast cancer and known meningioma presented with unilateral vision loss associated with anisocoria and an afferent pupillary defect. On magnetic resonance imaging we found the cause to be optic nerve compression by a right frontal meningioma. Monocular vision-loss etiologies are anatomically localized to structures anterior to the optic chiasm. This case serves as a reminder that cerebral structures in this location must not be forgotten in the differential.

## CASE PRESENTATION

**Image 1:**
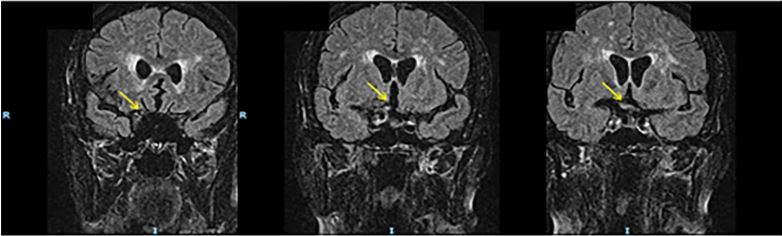
Coronal FLAIR (fluid-attenuated inversion recovery) demonstrating optic nerve compression (yellow arrow).

**Image 2:**
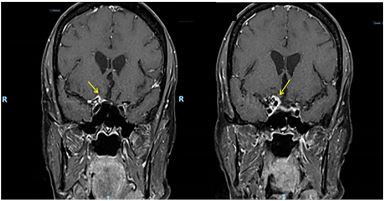
Transverse T2 image demonstrating frontal lobe mass abutting the optic nerve (yellow arrow).

Our patient had a past medical history significant for right frontal lobe meningioma and metastatic breast cancer who awoke from sleep approximately 20 hours prior to presentation with acute, painless, right eye vision loss and enlarged right pupil. Her physical exam consisted of visual acuities of 20/100 right, 20/25 left, right pupillary enlargement with afferent pupillary defect, and normal ocular pressure bilaterally. Magnetic resonance imaging (MRI) demonstrates pre-chiasmatic optic nerve compression secondary to frontal lobe mass (Images 1 and 2).

## DISCUSSION

The visual pathway requires that causes of monocular vision loss sit anterior to the optic chiasm.^1^ One must be mindful of the cerebral anatomy within the cranium, where the frontal lobe sits anterior to the optic chiasm. Therefore, mass effect must be considered in the differential for monocular vision loss. Prior to MRI, we obtained a computed tomography for definitive imaging secondary to previously known meningioma measuring 1.7 centimeters by 2 centimers.

**CPC-EM Capsule**

What do we already know about this clinical entity?

*Acute vision loss requires prompt evaluation with a careful history and physical examination. Due to the nerve and vascular supply to the eye, the etiologies of monocular vision loss are most commonly secondary to vascular, intra-ophthalmic, or inflammatory pathologies*.

What is the major impact of the image(s)?

*The magnetic resonance image in this report demonstrates a frontal lobe mass that impinges on the optic nerve, a rare cause of monocular vision loss*.

How might this improve emergency medicine practice?

*This case report can remind emergency physicians that, in addition to ophthalmologic and vascular causes, monocular vision loss can be caused by central nervous system lesions that are located anterior to the optic chiasm*.^2^
